# 4,4′-Bis(1,2,4-triazol-1-ylmeth­yl)biphen­yl

**DOI:** 10.1107/S1600536809038604

**Published:** 2009-09-30

**Authors:** Jianjun Xu

**Affiliations:** a Mechanical and Electrical Engineering Institute, North University of China, Taiyuan, 030051, People’s Republic of China

## Abstract

In the title compound, C_18_H_16_N_6_, the complete mol­ecule is generated by crystallographic inversion symmetry. The dihedral angle between the benzene and triazole rings is 84.1 (3)°. The crystal structure is stabilized by weak C—H⋯N hydrogen bonds.

## Related literature

For a related structure, see: Wang *et al.* (2007 ▶). For background to the use of flexible ligands to form coordination networks, see: Martin *et al.* (2007 ▶); Yaghi *et al.* (1998 ▶); Sun *et al.* (2006 ▶).
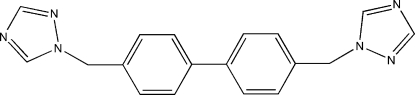


## Experimental

### 

#### Crystal data

C_18_H_16_N_6_
*M* *_r_* = 316.37
Monoclinic, 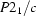

*a* = 16.590 (3) Å
*b* = 5.3646 (9) Å
*c* = 8.8009 (14) Å
β = 92.567 (3)°
*V* = 782.5 (2) Å^3^
*Z* = 2
Mo *K*α radiation
μ = 0.09 mm^−1^
*T* = 298 K
0.21 × 0.17 × 0.11 mm

#### Data collection

Bruker APEXII area-detector diffractometer
Absorption correction: multi-scan (*SADABS*; Bruker, 2005 ▶) *T*_min_ = 0.982, *T*_max_ = 0.991
3679 measured reflections
1402 independent reflections
870 reflections with *I* > 2σ(*I*)
*R*_int_ = 0.023

#### Refinement

*R*[*F* ^2^ > 2σ(*F* ^2^)] = 0.042
*wR*(*F* ^2^) = 0.107
*S* = 0.83
1402 reflections
109 parameters
H-atom parameters constrained
Δρ_max_ = 0.12 e Å^−3^
Δρ_min_ = −0.13 e Å^−3^

### 

Data collection: *APEX2* (Bruker, 2005 ▶); cell refinement: *SAINT* (Bruker, 2005 ▶); data reduction: *SAINT*; program(s) used to solve structure: *SHELXS97* (Sheldrick, 2008 ▶); program(s) used to refine structure: *SHELXL97* (Sheldrick, 2008 ▶); molecular graphics: *ORTEP-3* (Farrugia, 1997 ▶) and *PLATON* (Spek, 2009 ▶); software used to prepare material for publication: *SHELXL97*.

## Supplementary Material

Crystal structure: contains datablocks I, global. DOI: 10.1107/S1600536809038604/hb5117sup1.cif

Structure factors: contains datablocks I. DOI: 10.1107/S1600536809038604/hb5117Isup2.hkl

Additional supplementary materials:  crystallographic information; 3D view; checkCIF report

## Figures and Tables

**Table 1:** Hydrogen-bond geometry (Å, °)

*D*—H⋯*A*	*D*—H	H⋯*A*	*D*⋯*A*	*D*—H⋯*A*
C2—H2⋯N1^i^	0.93	2.56	3.381 (2)	148

Symmetry code: (i) 
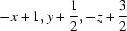
.

**Fig. 1.**
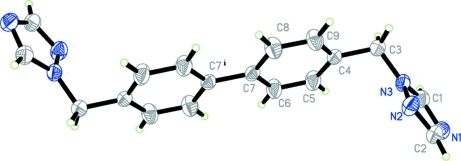
View of (I), showing ellipsoids drawn at the the 30% probability level. H atoms are shown as spheres of arbitrary radius. Symmetry code: (i) –x, 1–y, 1–z.

## supplementary crystallographic information

### Comment

It is well-known that those ligands containing a flexible backbone provide a bigger number of complexes thanks to their flexibility and conformational freedom that allow for greater structural diversity (Yaghi *et al.*,  1998; Sun *et al.*, 2006; Martin *et al.*,  2007).

4,4'-Bis(1,2,4-triazol-1-ylmethyl)biphenyl (bix) is a excellent building block and has been employed to construct interesting structural polymer with unique properties (Wang *et al.*,  2007).

In an attempt to form a Zn(II) complex with bix, we adventitiously formed the title compound (I) and its crystal structure is determined herein.

The title compound cyrstallizes with one half-molecule in the asymmetric unit. As illustrated in Fig. 1, the bix adopts a anti conformation and has crystallographic 1 symmetry and the dihedral angle between the benzene and triazole rings is 84.1 (3)°.

In the crystal structure, weak intermolecular C—H···N hydrogen bond help to stabilizing the packing.

### Experimental

Equimolar (28 mg, 0.1 mmol) Zn(OAC)_2_.6H_2_O in water (3 ml) and bix (26 mg, 0.1 mmol) in CH_3_CN and CH3OH solutions (8 ml) were mixed and heated at 428 K for 72 h in a pressurized reactor. Slow evaporation of this solution resulted in the formation of some colourless blocks of (I).

### Refinement

All H atoms were fixed geometrically and treated as riding with C—H = 0.93 Å  and U_iso_(H) = 1.2U_eq_(C).

### Crystal data

**Table:**

C_18_H_16_N_6_	*F*(000) = 332
*M**_r_* = 316.37	*D*_x_ = 1.343 Mg m^−^^3^
Monoclinic, *P*2_1_/*c*	Mo *K*α radiation, λ = 0.71073 Å
Hall symbol: -P 2ybc	Cell parameters from 1402 reflections
*a* = 16.590 (3) Å	θ = 2.5–25.2°
*b* = 5.3646 (9) Å	µ = 0.09 mm^−^^1^
*c* = 8.8009 (14) Å	*T* = 298 K
β = 92.567 (3)°	Block, colourless
*V* = 782.5 (2) Å^3^	0.21 × 0.17 × 0.11 mm
*Z* = 2	

### Data collection

**Table:**

Bruker APEXII area-detector diffractometer	1402 independent reflections
Radiation source: fine-focus sealed tube	870 reflections with *I* > 2σ(*I*)
graphite	*R*_int_ = 0.023
φ and ω scans	θ_max_ = 25.2°, θ_min_ = 2.5°
Absorption correction: multi-scan (*SADABS*; Bruker, 2005)	*h* = −19→18
*T*_min_ = 0.982, *T*_max_ = 0.991	*k* = −6→5
3679 measured reflections	*l* = −10→10

### Refinement

**Table:**

Refinement on *F*^2^	Primary atom site location: structure-invariant direct methods
Least-squares matrix: full	Secondary atom site location: difference Fourier map
*R*[*F*^2^ > 2σ(*F*^2^)] = 0.042	Hydrogen site location: inferred from neighbouring sites
*wR*(*F*^2^) = 0.107	H-atom parameters constrained
*S* = 0.83	*w* = 1/[σ^2^(*F*_o_^2^) + (0.05*P*)^2^ + 0.3476*P*] where *P* = (*F*_o_^2^ + 2*F*_c_^2^)/3
1402 reflections	(Δ/σ)_max_ < 0.001
109 parameters	Δρ_max_ = 0.12 e Å^−^^3^
0 restraints	Δρ_min_ = −0.13 e Å^−^^3^

### Special details

**Table:**

Geometry. All esds (except the esd in the dihedral angle between two l.s. planes) are estimated using the full covariance matrix. The cell esds are taken into account individually in the estimation of esds in distances, angles and torsion angles; correlations between esds in cell parameters are only used when they are defined by crystal symmetry. An approximate (isotropic) treatment of cell esds is used for estimating esds involving l.s. planes.
Refinement. Refinement of F^2^ against ALL reflections. The weighted R-factor wR and goodness of fit S are based on F^2^, conventional R-factors R are based on F, with F set to zero for negative F^2^. The threshold expression of F^2^ > 2sigma(F^2^) is used only for calculating R-factors(gt) etc. and is not relevant to the choice of reflections for refinement. R-factors based on F^2^ are statistically about twice as large as those based on F, and R- factors based on ALL data will be even larger.

### Fractional atomic coordinates and isotropic or equivalent isotropic displacement parameters (Å^2^)

**Table:**

	*x*	*y*	*z*	*U*_iso_*/*U*_eq_	
N3	0.34746 (8)	0.4361 (3)	0.45637 (17)	0.0522 (4)	
C7	0.04239 (10)	0.4897 (3)	0.4755 (2)	0.0465 (5)	
C4	0.20243 (10)	0.4473 (4)	0.3827 (2)	0.0511 (5)	
C3	0.28751 (10)	0.4191 (4)	0.3315 (2)	0.0602 (6)	
H3A	0.2927	0.2590	0.2817	0.072*	
H3B	0.2979	0.5479	0.2576	0.072*	
N2	0.35680 (10)	0.6499 (3)	0.5390 (2)	0.0651 (5)	
C2	0.41506 (12)	0.5892 (5)	0.6378 (2)	0.0656 (6)	
H2	0.4352	0.7001	0.7113	0.079*	
N1	0.44371 (10)	0.3558 (4)	0.6256 (2)	0.0680 (5)	
C6	0.09257 (11)	0.2995 (5)	0.5269 (3)	0.0714 (7)	
H6	0.0733	0.1827	0.5945	0.086*	
C1	0.39952 (12)	0.2674 (4)	0.5101 (2)	0.0605 (6)	
H1	0.4042	0.1071	0.4713	0.073*	
C9	0.15350 (12)	0.6357 (4)	0.3306 (3)	0.0673 (6)	
H9	0.1732	0.7527	0.2638	0.081*	
C8	0.07478 (12)	0.6559 (4)	0.3758 (3)	0.0682 (6)	
H8	0.0428	0.7858	0.3374	0.082*	
C5	0.17066 (11)	0.2789 (5)	0.4804 (3)	0.0718 (7)	
H5	0.2025	0.1471	0.5165	0.086*	

### Atomic displacement parameters (Å^2^)

**Table:**

	*U*^11^	*U*^22^	*U*^33^	*U*^12^	*U*^13^	*U*^23^
N3	0.0403 (8)	0.0588 (10)	0.0573 (9)	−0.0008 (8)	0.0015 (7)	−0.0034 (8)
C7	0.0429 (9)	0.0495 (11)	0.0467 (10)	−0.0041 (8)	−0.0038 (8)	−0.0044 (9)
C4	0.0423 (10)	0.0637 (13)	0.0470 (10)	−0.0044 (9)	−0.0030 (8)	−0.0056 (10)
C3	0.0468 (11)	0.0801 (15)	0.0535 (11)	−0.0024 (10)	0.0002 (9)	−0.0071 (11)
N2	0.0543 (10)	0.0649 (12)	0.0754 (12)	−0.0021 (9)	−0.0055 (8)	−0.0133 (10)
C2	0.0507 (11)	0.0816 (17)	0.0640 (13)	−0.0115 (12)	−0.0022 (10)	−0.0072 (12)
N1	0.0562 (10)	0.0846 (14)	0.0627 (11)	0.0017 (10)	−0.0025 (8)	0.0150 (10)
C6	0.0494 (12)	0.0846 (17)	0.0804 (15)	0.0065 (11)	0.0063 (10)	0.0323 (13)
C1	0.0572 (12)	0.0605 (13)	0.0640 (13)	0.0020 (10)	0.0057 (10)	0.0072 (11)
C9	0.0586 (12)	0.0620 (14)	0.0826 (15)	0.0013 (11)	0.0168 (11)	0.0155 (12)
C8	0.0592 (13)	0.0582 (13)	0.0880 (16)	0.0095 (11)	0.0140 (11)	0.0177 (12)
C5	0.0474 (12)	0.0834 (17)	0.0843 (16)	0.0113 (11)	0.0012 (10)	0.0264 (13)

### Geometric parameters (Å, °)

**Table:**

N3—C1	1.324 (2)	N2—C2	1.312 (3)
N3—N2	1.363 (2)	C2—N1	1.345 (3)
N3—C3	1.451 (2)	C2—H2	0.9300
C7—C8	1.377 (3)	N1—C1	1.315 (3)
C7—C6	1.380 (3)	C6—C5	1.380 (3)
C7—C7^i^	1.494 (3)	C6—H6	0.9300
C4—C9	1.363 (3)	C1—H1	0.9300
C4—C5	1.369 (3)	C9—C8	1.386 (3)
C4—C3	1.508 (3)	C9—H9	0.9300
C3—H3A	0.9700	C8—H8	0.9300
C3—H3B	0.9700	C5—H5	0.9300
			
C1—N3—N2	109.13 (15)	N1—C2—H2	122.3
C1—N3—C3	129.93 (18)	C1—N1—C2	102.16 (18)
N2—N3—C3	120.94 (16)	C7—C6—C5	121.5 (2)
C8—C7—C6	116.16 (17)	C7—C6—H6	119.3
C8—C7—C7^i^	122.4 (2)	C5—C6—H6	119.3
C6—C7—C7^i^	121.5 (2)	N1—C1—N3	111.2 (2)
C9—C4—C5	117.42 (18)	N1—C1—H1	124.4
C9—C4—C3	121.69 (19)	N3—C1—H1	124.4
C5—C4—C3	120.87 (19)	C4—C9—C8	121.1 (2)
N3—C3—C4	112.74 (15)	C4—C9—H9	119.5
N3—C3—H3A	109.0	C8—C9—H9	119.5
C4—C3—H3A	109.0	C7—C8—C9	122.1 (2)
N3—C3—H3B	109.0	C7—C8—H8	118.9
C4—C3—H3B	109.0	C9—C8—H8	118.9
H3A—C3—H3B	107.8	C4—C5—C6	121.7 (2)
C2—N2—N3	101.99 (17)	C4—C5—H5	119.1
N2—C2—N1	115.49 (19)	C6—C5—H5	119.1
N2—C2—H2	122.3		

Symmetry codes: (i) −*x*, −*y*+1, −*z*+1.

### Hydrogen-bond geometry (Å, °)

**Table:**

*D*—H···*A*	*D*—H	H···*A*	*D*···*A*	*D*—H···*A*
C2—H2···N1^ii^	0.93	2.56	3.381 (2)	148

Symmetry codes: (ii) −*x*+1, *y*+1/2, −*z*+3/2.
